# From Desert Greening to Human Health: A Systematic Review of the Extraction, Unique Structure, and Bioactivity of Sea Buckthorn Proanthocyanidins

**DOI:** 10.3390/foods14183203

**Published:** 2025-09-15

**Authors:** Zixin Zhou, Zongyi He, Yu He, Qiang Peng

**Affiliations:** College of Food Science and Engineering, Northwest A&F University, Yangling, Xianyang 712100, China

**Keywords:** sea buckthorn proanthocyanidins, extraction methods, structural characteristics, biological activities, industrial applications

## Abstract

Sea buckthorn (*Hippophae rhamnoides*) is an ecologically significant shrub that has been effectively employed to combat desertification. Recently, its economic potential has garnered substantial scientific interest. Sea buckthorn proanthocyanidins (SBPs), which are key antioxidant bioactive compounds found in sea buckthorn, exhibit remarkable free radical scavenging capacity and multi-target biological activities, including anti-inflammatory, anti-cancer, vision protection, and cardiovascular protection effects. These attributes highlight the potential applications of SBPs in functional foods, nutritional supplements, and skincare products. This review summarizes the current state of research regarding extraction methods, structural characteristics, and associated biological activities of sea buckthorn proanthocyanidins, while also exploring future research directions to provide a reference for their further development and utilization.

## 1. Introduction

Sea buckthorn (*Hippophae rhamnoides*), a member of the *Elaeagnaceae* family, is a deciduous shrub or small tree native to Asia and Europe. Due to its superior ecological benefits and economic advantages, it is widely cultivated artificially in China, particularly in provinces such as Gansu, Qinghai, Inner Mongolia, and Sichuan [1]. As a pioneer species in ecological restoration, sea buckthorn is highly adaptable and able to withstand a range of abiotic stresses, including drought, wind, sand, saline-alkali conditions, and poor soil. Its root system is notably well-developed, containing root nodules that facilitate nitrogen fixation, thereby enriching the soil and mitigating the effects of erosion, which contributes to the control of desertification [2,3]. Moreover, sea buckthorn fruit is a traditional Chinese medicinal herb with a long history of use in treating various ailments, including asthma, diabetes, gastric ulcers, cardiovascular disease, inflammation, and metabolic disorders. Ancient texts such as the Tang Dynasty’s *Yue Wang Yao Zhen*, the Tibetan medical classic *Si Bu Yi Dian*, and the Ming Dynasty’s *Compendium of Materia Medica* document its pharmacological activity. In 1977, the Chinese Ministry of Health included sea buckthorn in the first group of plants recognized for its medicinal and edible properties, further affirming its dual value in the food and pharmaceutical sectors. Modern research has demonstrated that sea buckthorn fruits are rich in a diverse array of active substances, including phenolic compounds, vitamins, flavonoids, polysaccharides, unsaturated fatty acids, carotenoids, and essential amino acids. These ingredients provide sea buckthorn with antioxidant, anti-inflammatory, immunomodulatory, circulatory-promoting, antibacterial, and other beneficial activities [4]. In addition to its medicinal applications, sea buckthorn serves as a versatile ingredient in various food products, such as juice, jam, tea, and soft candy, which possess significant nutritional value and economic benefits.

Proanthocyanidins are a class of polyphenolic compounds formed by the polymerization of flavan-3-ol monomer. They are widely distributed in 35 different plant families and 60 plant parts [5], including sea buckthorn, grapes, blueberries, cranberries, hazelnuts, sorghum, and lotus, making them a regular component of the human diet [6]. Proanthocyanidins have a defensive role in plants against biological stress, including microbial pathogens and insect pests [7]. In recent years, proanthocyanidins have garnered significant attention from the scientific community due to their potential health benefits and diverse range of applications. Studies have demonstrated that proanthocyanidins possess a variety of pharmacological activities, including antioxidant [8], anti-inflammatory [9], and antibacterial properties [10], and can contribute to the prevention of cardiovascular disease [11], cancer [12], and neurodegenerative diseases [13]. Consequently, proanthocyanidins have become the subject of considerable interest in the development of functional foods and dietary supplements. For instance, proanthocyanidin-rich grape seed extract is employed as an antioxidant supplement [14]. The utilization of proanthocyanidins in cosmetics is a subject of considerable research interest, with studies demonstrating the enhancement of superoxide dismutase (SOD) activity in skin fibroblasts and the promotion of skin health by proanthocyanidins [15]. Market analysis has revealed that the proanthocyanidin industry is experiencing rapid growth, in particular in the domains of cardiovascular health, anti-aging, and cancer prevention. A 2024 report highlighted the Asia-Pacific region, including countries such as China and India, as a significant market for proanthocyanidins due to the escalating demand for preventive healthcare. An increase in commercial interest has further facilitated the undertaking of scientific research in this field.

Proanthocyanidins are natural antioxidant compounds particularly abundant in sea buckthorn. According to Yang et al. [16], total proanthocyanidin content varies widely from 390 to 1940 mg/100 g of dry weight (DW), a variation heavily influenced by the interaction between subspecies and growth location. Notably, the upper limit of this range significantly exceeds the average content reported for many common fruits, such as peaches (approx. 300 mg/100 g DW) and apples (approx. 600 mg/100 g DW). Investigating different sea buckthorn proanthocyanidins in different parts of the plant, Tkacz et al. [17] reported a proanthocyanidin content from 141.25 to as high as 7275.98 mg/100 g DW. Proanthocyanidin concentrations increased in the order of flesh < endocarp < skins < leaves < seeds < branches, indicating that industrial by-products such as branches and seeds are exceptionally concentrated sources of proanthocyanidins. Furthermore, sea buckthorn proanthocyanidins (SBPs) exhibit notable antioxidant activity, which principally stems from their relatively rare monomeric composition characterized by a high degree of hydroxylation [18]. This structural feature confers a dual antioxidant capacity: the abundant hydroxyl groups function as direct radical scavengers to mitigate oxidative stress and simultaneously act as chelating agents for pro-oxidant metal ions, such as iron and copper, thereby inhibiting their catalytic involvement in oxidative reactions [19]. Modern research has demonstrated that SBPs offer multiple benefits for human health, including improvements in cardiovascular health [20], anti-cancer properties [21], eye protection [22], and enhanced skin health [15].

The convergence of global health crises—marked by escalating incidences of cardiovascular diseases, diabetes, and age-related degenerative disorders—and mounting ecological challenges, including desertification and biodiversity loss, suggests the pressing need for integrated strategies that reconcile human health with environmental sustainability [3]. Influenced by an evolving focus on global health priorities from disease-centric treatment to prevention-oriented approaches, the market for functional foods and multipurpose dietary supplements has expanded rapidly. Plant-derived bioactive compounds, particularly those with reported safety profiles and multi-target health benefits, are increasingly being considered promising candidates for such applications [14]. Within this context, sea buckthorn may be regarded as a noteworthy phytochemical resource, potentially offering avenues to address both ecosystem restoration and preventive healthcare [2,4]. As a keystone species in arid land rehabilitation, sea buckthorn not only helps stabilize fragile ecosystems but also synthesizes a suite of multifunctional bioactive metabolites. This higher degree of B-ring hydroxylation is a key structural feature that is thought to underpin its potent and unique bioactivity profile, encompassing antioxidant, anti-inflammatory, and metabolic regulatory effects [18]. However, current research has predominantly focused on traditional proanthocyanidin sources, leaving the ecological–pharmacological interactions of SBPs less thoroughly explored. This knowledge gap hinders the full utilization of SBPs for integrated health-environment solutions.

To bridge these gaps, this review systematically synthesizes the existing literature. The methodology followed the principles of a systematic review, involving a comprehensive search of scientific databases (including Scopus, Web of Science, PubMed, and Google Scholar) to identify all directly relevant studies, encompassing both recent advancements and seminal earlier works critical for foundational understanding. Search terms included “sea buckthorn proanthocyanidins,” “Hippophae rhamnoides,” “extraction,” “structural characterization,” and “biological activity.” Inclusion criteria mandated peer-reviewed original research articles in English that focused directly on SBPs, while review articles and studies on other sea buckthorn components were excluded to maintain focus. Building on this curated body of literature, this review aims to (i) explore advancements in SBP extraction, structural characterization, and bioactivity validation; (ii) examine its potential industrial applications in nutraceuticals, cosmeceuticals, and ecological products; and (iii) suggest possible future research directions to help optimize SBP utilization.

## 2. Preparation of SBPs

**Figure 1:**
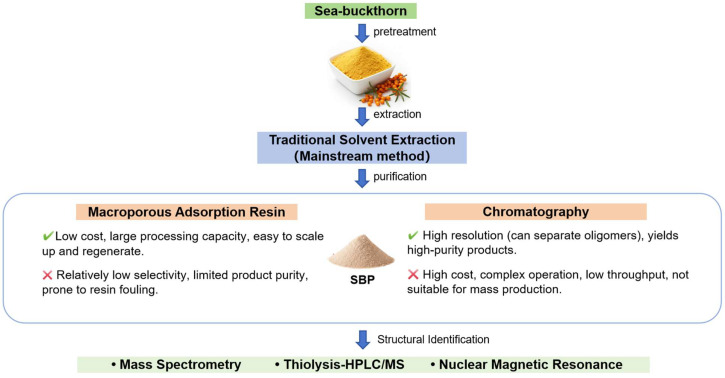
The process of SBP preparation and structural analysis.

The general procedure for preparing SBPs involves pretreatment, extraction of SBPs, and subsequent purification steps. Pretreatment includes raw material selection, cleaning, drying, and grinding, which not only standardizes the biomass but also effectively improves the extraction efficiency due to changes such as cell fragmentation, increased surface area, obstacle removal, and phase change [23]. The commonly reported extraction methods for SBPs currently primarily feature the traditional solvent method. The choice of solvent and extraction conditions, including temperature, solvent-to-biomass ratio, and extraction time, plays a crucial role in determining the chemical properties and functionality of the extracted SBPs. The purification of the extracted proanthocyanidins is an effective process for the removal of impurities, including sugars, organic acids, and other phenolic compounds. The selection of the purification method depends on various factors, such as the desired purity of the product, the operational scale, the pH of the medium, and the concentration of the extract. Representative techniques include chromatography [24] and resin adsorption. Figure 1 provides a visual roadmap of this entire process, critically comparing the advantages and disadvantages of different purification strategies. For a detailed comparative overview, the key parameters, source materials, and resulting purities from representative studies have been systematically compiled in Table 1.

### 2.1. Extraction of SBPs

Currently, research on the extraction of proanthocyanidins from sea buckthorn primarily focuses on traditional solvent extraction methods. The solvent extraction method relies on polarity-driven selective dissolution between the solvent and proanthocyanidin molecular structure. Extraction efficiency is predominantly governed by five parameters: solvent selection, solid-to-liquid ratio, temperature, pH, and extraction time [31]. Proanthocyanidins exhibit strong polarity due to their polyphenolic hydroxyl groups, necessitating polar solvents such as water, acetone, and ethanol for optimal recovery. Xu et al. [25] investigated a new technology for the extraction of proanthocyanidins derived from sea buckthorn bark. They found the solvent efficiency ranked in the order of methanol > acetone > ethanol > water. Although water is a suitable solvent for polyphenols, the extraction yield was low because SBPs are bound to the abundant plant matrix in the bark (e.g., cellulose) via hydrogen and hydrophobic bonds, and water has difficulty disrupting this binding, which consequently compromises extraction efficiency. Therefore, comprehensively considering solvent toxicity and environmental friendliness, they ultimately selected ethanol for further optimization. Through single-factor experiments, they determined that the optimal conditions for the SBP extraction curve were a temperature of 20 °C, an extraction time of 90 min, a pH of 4.80, a solid-to-liquid ratio of 1:10 (g/mL), and an ethanol concentration of 60%.

The acidic environment can facilitate the release of proanthocyanidins by degrading the pectin polysaccharides in the plant cell wall [32]. It is important to note that proanthocyanidins are susceptible to conversion into anthocyanins under acidic conditions and elevated temperatures [33]. Consequently, the use of acidic acetone for extracting proanthocyanidins requires meticulous regulation of pH and temperature. By comparing aqueous methanol and acetone solutions with varying concentrations and acid addition conditions, Arimboor et al. [27] determined the optimal solvent system for extracting proanthocyanidins from sea buckthorn pulp, seed coat, kernel, and leaves. The results indicated that the acetone–water mixture containing 1% acid (70:30) was the most effective solvent for extracting proanthocyanidins from berry pulp and kernel. In contrast, the acetone-water mix with 1% acid (60:40) was optimal for sea buckthorn leaves and seed coats.

The solvent composition exerts a significant influence not only on the extraction efficiency of SBPs but also on the average degree of polymerization (ADP) of the extracted proanthocyanidins. Proanthocyanidins in sea buckthorn exhibit a wide range of degrees of polymerization (DP), with their polarity decreasing with increasing DP. Consequently, by adjusting the extraction solvent, it is possible to extract more oligomers or polymers selectively. Studies have shown that proanthocyanidins with low DP are more soluble in more polar solvents such as water or methanol. In contrast, proanthocyanidins with high DP are more suitable for less polar solvents such as acetone [34]. Under conditions that are identical to those employed in conventional extraction protocols, lower polarity acetone/water/acetic acid (70:29.5:0.5, *v*/*v*/*v*) extracts 67% and 77% less epicatechin and catechin, respectively, than higher polarity 50% ethanol/water (*v*/*v*) [33]. This demonstrates that increasing the polarity of the solvent improves the selectivity for lower DP proanthocyanidins. Chen et al. [35] used eluents with decreasing polarity to separate multiple batches of proanthocyanidins from grape seeds and pomace. They found that the ADP of proanthocyanidins increased over time, indicating that proanthocyanidins with lower ADP exhibit higher hydrophilicity and are eluted first. Additionally, Wang et al. [36] found that proanthocyanidins with higher concentrations of galloyl groups exhibit greater lipophilicity. This suggests that in addition to the degree of polymerization, other factors affecting molecular polarity are also worth considering. Selecting a solvent with moderate polarity allows for the acquisition of the maximum total proanthocyanidin yield. One study compared six ethanol/water solvent methods and found that 50% ethanol/water (*v*/*v*) extraction with moderate polarity extracted more total proanthocyanidins than other ethanol/water compositions [33]. Another way to obtain more proanthocyanidins is to carry out multiple extractions. For instance, in one study, pomace was extracted twice to obtain proanthocyanidins, with the initial extraction conducted at a ratio of 1 g/10 mL methanol: water (80:20, *v*/*v*) and the subsequent extraction utilizing acetone: water (75:25, *v*/*v*) at the same solid–liquid ratio, thereby enhancing the extraction of proanthocyanidins [37].

The temperature and duration of extraction are also significant factors that must be taken into consideration. The influence of temperature on SBP extraction is a critical but double-edged sword that requires careful optimization. On one hand, elevated temperatures can enhance extraction yield. For instance, Zhu et al. [30] investigated a green solvent extraction method to extract SBPs involving two hot water extractions (1:15 *m*/*v*, 55 °C, 4 h) from sea buckthorn powder, and the overall yield and purity were 9.1% and 91.5%, respectively. Temperature emerges as a key determinant for the favorable yield and purity, as the increased temperature tends to weaken or break hydrogen bonds. This reduces the binding strength between proanthocyanidins and the plant matrix, compensating to some extent for the drawbacks of using water as a solvent. On the other hand, excessive heat can be detrimental to the structural integrity of SBPs. A study using gel permeation chromatography revealed that extraction temperatures above 100 °C led to the depolymerization of proanthocyanidins, significantly reducing ADP [38]. Therefore, a clear trade-off exists: the temperature must be high enough to improve mass transfer but low enough to prevent thermal degradation. The optimal temperature is thus highly dependent on the specific solvent system and target product characteristics. Moreover, Lisjak et al. [39] investigated the effect of maceration time on the extraction of proanthocyanidins and found that the extraction of high molecular-weight proanthocyanidins from seeds increased for up to 20 days, whereas low molecular-weight proanthocyanidins reached a plateau earlier.

To date, the application of modern techniques such as ultrasound-assisted extraction (UAE) or microwave-assisted extraction (MAE) specifically for SBPs remains largely undocumented in the peer-reviewed literature. This represents a significant research gap. However, the potential of these technologies can be inferred from their success with other proanthocyanidin-rich materials. For example, UAE significantly enhanced proanthocyanidin yield from grape pomace, more than doubling the recovery compared to conventional methods [40]. Similarly, MAE drastically reduced extraction time for proanthocyanidins from grape pomace while maintaining high yields [41]. Given that SBP extraction faces challenges such as strong bonding to the plant matrix, these green technologies are highly promising for future investigation. Future studies should focus on optimizing UAE or MAE parameters for sea buckthorn to potentially increase yield, shorten extraction time, and reduce solvent consumption, thereby developing more sustainable SBP production methods.

### 2.2. Purification of SBPs

#### 2.2.1. Chromatography

The chromatographic process is characterized by its ability to achieve high levels of resolution, which is a prerequisite for the analysis of complex samples. The primary influencing factors of this method comprise the type of column packing, the composition of the eluent, and the flow rate. Additionally, it is imperative to consider the effects of pH and temperature on the stability of the extract [42]. Sephadex column chromatography is a common method for purifying SBPs. In the study of Arimboor et al. [27], 500 mg of crude proanthocyanidin extract was suspended in 95% (*v*/*v*) ethanol and loaded onto a Sephadex LH-20 column previously equilibrated with 95% ethanol (*v*/*v*). After this, the column was thoroughly flushed with 1 L of 95% ethanol (*v*/*v*), and then the crude extract was eluted with 1.5 L of 50% acetone until no proanthocyanidins were detected in the eluate, with a purity of 59.5% to 70.4%. Rösch et al. [18] used Sephadex LH-20 gel chromatography to isolate proanthocyanidins from sea buckthorn pomace. The column was washed with 400 mL of water, after which the methanol content of the eluent was increased from 0 to 100% (*v*/*v*) in 10% increments. Elution was continued with two portions of an acetone-water mixture (70:30, *v*/*v*), and the fractions were designated A–M. The relatively high purity of proanthocyanidins extracted by chromatography renders them suitable for laboratory research; however, their industrial application is limited by the high cost of dedicated columns and organic solvents. Recent advancements, such as the utilization of deep eutectic solvents (DES) as mobile phase modifiers, have the potential to enhance selectivity and reduce reliance on contaminating organic solvents [43].

High-performance liquid chromatography (HPLC) is another widely utilized chromatographic technique, primarily operating in two modes: normal-phase (NP) and reversed-phase (RP). NP-HPLC employs a polar stationary phase to separate proanthocyanidins based on their degree of polymerization. Compounds with higher polymerization degrees exhibit stronger interactions with the polar stationary phase, resulting in delayed elution. This mode is particularly effective for isolating extractable forms of proanthocyanidins. In contrast, RP-HPLC utilizes a non-polar stationary phase, separating specific oligomers according to their hydrophobicity. This approach is often coupled with complementary techniques, such as size-exclusion chromatography, to enhance separation efficacy. In the study by Adamson et al. [44], initial enrichment via Sephadex LH-20 gel permeation chromatography was followed by preparative NP-HPLC, which successfully isolated proanthocyanidins spanning from dimers to decamers. This outcome underscores the proficiency of NP-HPLC in resolving polymerization-dependent heterogeneity. To investigate the composition and structure of sea buckthorn seed proanthocyanidins, Fan et al. [26] conducted chromatographic separation of SBPs. The crude SBP-containing extract was loaded onto a Sephadex LH-20 column pre-equilibrated with water. The column was sequentially eluted with a water-ethanol gradient, where the ethanol concentration was increased stepwise by 10% increments (from 0% to 70%), followed by elution with a water–acetone mixture (3:7, *v*/*v*), yielding nine fractions. Due to the potentially greater diversity of configurations among the less polar, high-DP proanthocyanidins, the final fraction underwent further separation via Sephadex LH-20 column chromatography using a water-ethanol-acetone mobile phase. Subsequently, coupling with the thiolysis reaction and mass spectrometry allowed for the elucidation of the SBP polymers, including the composition of their extension and terminal flavan-3-ol units. Furthermore, Pasini employed Sephadex LH-20 column chromatography to fractionate proanthocyanidins, followed by NP-HPLC coupled with fluorescence detection and electrospray ionization mass spectrometry (ESI-MS) using a Develosil Diol 100Å column. This analytical approach enabled the identification of oligomeric proanthocyanidins ranging from monomers to dodecamers, thereby demonstrating the efficacy of HPLC in resolving phenolic compound distributions within botanical matrices [45]. HPLC is distinguished by two principal advantages in proanthocyanidin purification: high resolution, capable of differentiating structurally similar compounds like oligomeric isomers, and scalability, enabling seamless transition from analytical (µg-scale) to preparative (mg-scale) applications. Its versatility is further amplified by compatibility with diverse stationary phases and hyphenated detection systems. Nevertheless, the technique is constrained by high operational costs and methodological complexity, necessitating optimization of parameters. Furthermore, this technique faces challenges in separating non-extractable proanthocyanidins, which are covalently bound to plant matrices, and highly polymerized proanthocyanidins (DP ≥ 10), compared to simple oligomers, polymeric proanthocyanidins are considerably more challenging to separate using HPLC techniques because the number of possible isomers increases with the degree of polymerization. Therefore, purification techniques are often necessary to isolate fractions containing mixtures of these polymers [26]. Recent innovations, such as hydrophilic interaction liquid chromatography (HILIC) employing diol-modified phases, offer promising avenues to address these limitations [46].

Despite the widespread adoption of chromatographic techniques in industrial applications, particularly in food analysis and quality control, several inherent limitations persist that hinder their scalability and efficiency [47]. Conventional methods such as high-performance liquid chromatography with ultraviolet (UV) detection often exhibit inadequate resolution and sensitivity when processing complex food matrices, resulting in the co-elution of analytes and potential underestimation of phenolic concentrations. Gas chromatography, while effective for volatile compounds, is constrained to analytes that can be vaporized without decomposition, necessitating derivatization for non-volatile phenolics that may compromise sample integrity. Thin-layer chromatography provides limited resolution and is primarily suited for qualitative screening rather than precise quantification. Moreover, extensive sample preparation procedures, including extraction and purification, are labor-intensive and susceptible to variability, thereby reducing throughput and reproducibility in high-volume industrial settings. The substantial consumption of organic solvents in these processes raises environmental and sustainability concerns, contributing to higher operational costs and regulatory challenges [48]. Even advanced variants, including nano-liquid chromatography and multi-dimensional approaches, face barriers such as technical complexity, elevated equipment expenses, and difficulties in method transferability across facilities, underscoring the need for ongoing innovations to enhance practicality and alignment with green chemistry principles in industrial contexts.

Traditional column chromatography, while cost-effective for preliminary separations, lacks the resolution required to isolate individual oligomers due to its reliance on broad polarity gradients. Size-exclusion chromatography, though effective in segregating compounds by molecular weight, fails to resolve isomers with identical polymerization degrees. Consequently, HPLC emerges as the method of choice for small-scale purifications that require the precise isolation of specific proanthocyanidin species. It is usually combined with other purification methods and used as the final purification method to improve product purity further. Notably, HPLC can be further employed for structural elucidation and quantification of proanthocyanidins through synergistic integration with emerging chromatographic modalities and advanced detection technologies such as tandem mass spectrometry.

#### 2.2.2. Resin Adsorption

Purifying proanthocyanidins using macroporous resins is a cost-effective and scalable approach. The resins selectively adsorb target molecules through hydrophobic interactions and hydrogen bonds while excluding polar impurities, with selectivity influenced by molecular weight and polarity. Zhao et al. [49] evaluated five macroporous resins for adsorbing proanthocyanidins from *Rhodiola rosea* and identified AB-8 as the most effective; its adsorption behavior was consistent with the Langmuir model for monolayer adsorption. Under the optimal conditions they established, which were a sample concentration of 4 mg/mL, a flow rate of 1.5 BV/h, and elution with three-bed volumes of 50% ethanol, a product purity of up to 64% was obtained. Furthermore, Zhu et al. [30] purified a concentrated solution of SBPs using AB-8 macroporous adsorption resin, ultimately achieving high SBP yield and purity, which indicates that the method can be effectively applied for the extraction of sea buckthorn proanthocyanidins. Xu et al. [25] systematically evaluated the static and dynamic adsorption/desorption capacities of 16 resin types to optimize the purification conditions of proanthocyanidins derived from sea buckthorn bark. Their results identified resin D3520 as the most promising candidate, exhibiting a dynamic adsorption capacity of 20.30 ± 1.22 mg/mL for proanthocyanidins. Under static conditions, the adsorption capacity increased to 28.96 ± 1.31 mg/mL, with a relative desorption degree of 87.84 ± 0.34%.

Although purification based on macroporous resins presents an economically advantageous strategy for large-scale industrial applications, it nonetheless faces significant technical limitations during industrial scale-up [50]. Firstly, resin fouling stands as a critical challenge [51]. Impurities such as sugars, proteins, and polysaccharides present in plant crude extracts can irreversibly adsorb onto or physically obstruct resin pores, consequently leading to a decline in adsorption capacity over time, a shortened operational lifespan, and the subsequent demand for frequent and costly regeneration or replacement [52]. Secondly, the regeneration process itself can become a significant production bottleneck, not only consuming substantial volumes of solvents but also, incomplete regeneration can result in inconsistent product quality across batches [53]. Furthermore, the selectivity of macroporous resins is inherently limited, often leading to the co-adsorption of target compounds with other structurally similar phenolic compounds, thereby diminishing the purity of the final product and making it generally difficult to achieve HPLC-level high-purity products through this method [54]. To enhance purity, the incorporation of additional downstream refining steps becomes imperative, which inevitably increases both process complexity and production costs [55]. Finally, in large-scale industrial chromatography columns, uneven liquid distribution can induce a “channeling” phenomenon, wherein the feedstock bypasses a significant portion of the resin bed, consequently reducing mass transfer efficiency and the effective utilization of the adsorbent [56]. Therefore, despite the advantages of macroporous resin methods in terms of production cost and environmental friendliness, these technical bottlenecks collectively hinder their direct application in the development of high-purity and high-value-added products [57].

Macroporous resin-based purification offers an economically favorable strategy for large-scale industrial applications. When coupled with eco-friendly eluents like ethanol, this technique demonstrates enhanced environmental compatibility compared to conventional purification processes that require extensive organic solvent consumption. Nevertheless, the resultant proanthocyanidin preparations typically exhibit lower purity levels than those obtained via HPLC, rendering this methodology particularly suited for industrial applications where ultrahigh purity is nonessential.

## 3. Structure and Identification Methods

**Figure 2:**
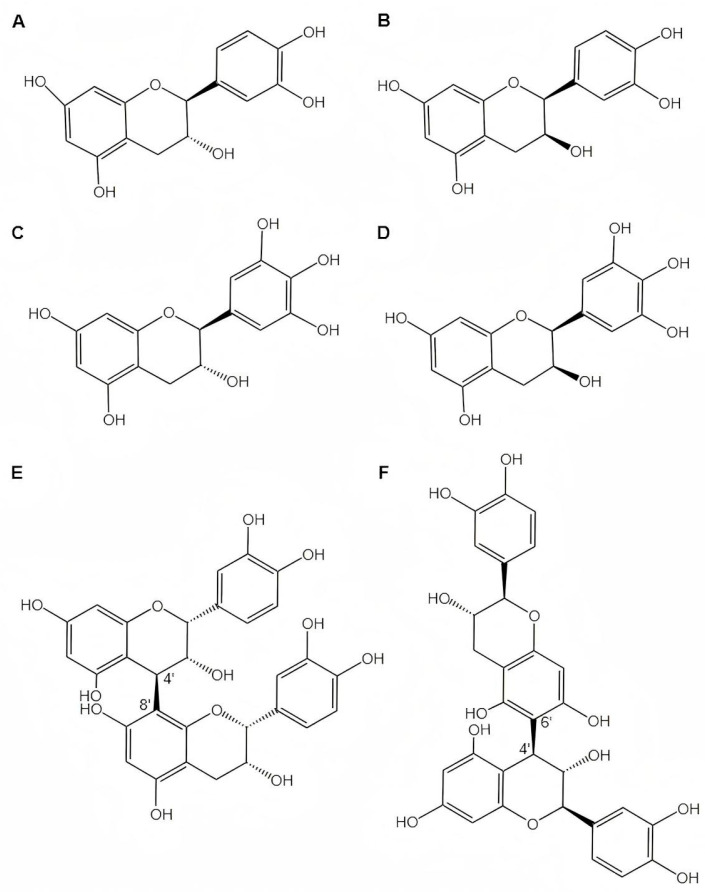
Monomeric structure and linkage patterns of SBPs. (**A**) catechin; (**B**) epicatechin; (**C**) gallocatechin; (**D**) epigallocatechin; (**E**) Typical C4 → C8 linkage pattern (exemplified by Proanthocyanidin B2); (**F**) Typical C4 → C6 linkage pattern (exemplified by Proanthocyanidin B6).

Proanthocyanidins are constructed from interconnected flavan-3-ol monomers. Due to differences in the type, order, and type of chemical bond connecting the monomers, as well as differences in the DP, spatial configuration, and kind of hydroxyl substitution, the proanthocyanidin composition in plants is highly complex and exists as a mixture [58]. The physicochemical properties and biological functions of proanthocyanidins are affected by the structures they possess. Figure 2 illustrates the structures of the flavan-3-ol monomeric units and their linkages in SBPs.

### 3.1. Monomer Composition

The structural basis of SBPs is the flavan-3-ol monomer unit, which mainly includes catechin (C), epicatechin (EC), gallocatechin (GC), and epigallocatechin (EGC) [28]. These monomeric subunits exhibit differential hydroxylation patterns and stereochemical configurations, providing the molecular basis for the conformational diversity of proanthocyanidins. Notably, the free radical scavenging capacity of the compounds decreases in the following order: EGC > GC > EC > C [59]. One of the influencing factors is that C and EC have a dihydroxylated B-ring, whereas GC and EGC have a trihydroxylated B-ring with an additional hydroxyl group, giving them greater antioxidant capacity. Sea buckthorn is a rare plant that uses GC and EGC as the main monomers, and GC subunits are only found in significant proportions in the proanthocyanidins of the toxic plant *Iris pseudacorus* [18]. This special composition of monomers with high free radical scavenging capacity helps sea buckthorn respond to harsh environments, indicating that sea buckthorn may have evolved unique secondary metabolic pathways to adapt to stressful environments.

### 3.2. Linkage Mode and Stereochemistry

The intermolecular linkages of SBPs are principally C4 → C8, with a minor number of C4 → C6 linkages, which is characteristic of proanthocyanidins of type B [24]. Nuclear magnetic Resonance analysis revealed a key stereochemical feature: more than 75% of the flavan-3-ol subunits in SBPs exist in the 2,3-trans configuration. This is in sharp contrast to the 2,3-cis configuration that predominates in common sources like apple proanthocyanidins [18]. The importance of this structural distinction is significant for SBPs’ bioactivity. The 2,3-trans arrangement imparts a more planar molecular geometry, which is hypothesized to directly contribute to SBPs’ potent antioxidant capacity. This enhanced planarity facilitates greater electron delocalization across the molecule’s conjugated system. Consequently, the radical formed after scavenging a free radical is better stabilized, increasing the overall efficiency of the antioxidant action. This unique stereochemistry is therefore a critical factor underlying SBPs’ distinct chemical behavior and bioactivity profile.

### 3.3. Degree of Polymerization

This variability represents a point of divergence in the literature. For instance, while some analyses suggest that high-DP polymers constitute the bulk of SBPs [24], other studies using different extraction and analysis techniques report a predominance of oligomers [18,30].

These conflicting findings are not necessarily contradictory; rather, they highlight that SBPs’ DP is influenced by a combination of factors. These include the extraction methodology, plant variety, growth environment, and importantly, the specific plant tissue analyzed. This tissue-dependent variation is particularly pronounced, with SBPs from leaves exhibiting the highest ADP (10.6), followed by the seed coat (8.2), flesh (7.4), and seed kernel (5.6) [27]. This structural difference serves a clear ecological purpose: the highly polymerized proanthocyanidins in leaves offer enhanced chemical defense against herbivores by more effectively precipitating digestive enzymes. Therefore, understanding the DP of an SBP sample requires careful consideration of its origin and processing history.

### 3.4. The Influence of Geographical and Climatic Factors

The genetic background of a plant is the primary determinant of the proanthocyanidins in plant tissues, but environmental factors can also influence their composition. Yang et al. [60] used bivariate correlation analysis to investigate the relationship between weather variables and proanthocyanidin composition in two distinct varieties of sea buckthorn berries. They found that the total SBP level was negatively correlated with the majority of temperature-related variables but positively correlated with most precipitation and humidity variables. The researchers hypothesize that this phenomenon is attributable to the capacity of sea buckthorn to sustain elevated photosynthetic rates at low temperatures, thereby facilitating the synthesis of substantial quantities of secondary metabolites. Another study has indicated that the total proanthocyanidin content in high-altitude populations is twice that observed in low-altitude populations, with a concomitant increase in the proportion of high-DP polymers (DP ≥ 5). This phenomenon has been linked to UVB-induced oxidative stress, with high DP polymers potentially functioning as UV filters and free radical scavengers to protect sea buckthorn from environmental damage [16]. Furthermore, high-altitude populations exhibited a higher GC ratio, which is attributable to the upregulation of white anthocyanin reductase by cold stress, promoting the biosynthesis of GC and EGC. This process contributes to the stabilization of cell membranes, enabling adaptation to low temperatures. This change reflects the importance of SBPs in environmental adaptation. This demonstrated metabolic plasticity may also represent a significant adaptive advantage in the context of global climate change. As regional weather patterns shift, the ability of sea buckthorn to modulate its proanthocyanidin profile in response to variables like precipitation and humidity could enhance its resilience and survival. This capacity to reinforce its chemical defenses against environmental stressors further underscores its value as a robust and adaptable species for ecological restoration in challenging and changing environments.

### 3.5. Structure–Activity Relationship

In addition to their unique monomeric composition, the DP differences of SBPs are also important factors affecting their antioxidant activity and physiological activity. Studies have shown that oligomers (DP 2–4) have stronger free radical scavenging ability due to the exposure of more phenolic hydroxyl groups. For instance, SBPs with a low DP are more effective at scavenging superoxide anion (ADP ≤ 4.2) and hydroxyl radicals (ADP ≤ 5.9). The antioxidant activity of polymers (DP ≥ 5) decreases with increasing DP, which may be related to the decrease in hydroxyl active sites due to steric hindrance, but polymers demonstrate superior chelating ability for metal ions, such as Fe^3+^. However, this DP-dependent activity is not universally observed across all antioxidant assays. Although SBP oligomers are effective scavengers of superoxide anions, their degree of polymerization does not significantly correlate with specific antioxidant indicators, such as iron-reducing capacity, DPPH, and ABTS radical scavenging [28]. The underlying mechanism for this discrepancy warrants further investigation.

In terms of biological activity, while specific human bioavailability data for SBP oligomers versus polymers remains scarce, insights from the broader proanthocyanidin literature suggest that oligomers have a higher oral bioavailability, can penetrate cell membranes, are directly absorbed in the small intestine, and then directly act on the target sites in the body, so oligomers are the primary source of biological activity. Conversely, high-molecular-weight compounds encounter difficulties with direct absorption in the intestine, predominantly undergoing metabolic transformation by colonic microorganisms into phenylpropionic acid and phenyl γ-valerolactone metabolites. These, in turn, exert indirect anti-inflammatory effects by modulating intestinal flora [61]. Confirming this differential metabolic model specifically for unique (epi)gallocatechin-rich SBPs stands as a critical area for future investigation.

### 3.6. Structural Identification Methods

Elucidating the complex structures of SBPs, as detailed in the preceding sections, requires a suite of powerful analytical techniques. To provide a clear, comparative analysis of their respective capabilities, the principles, key information provided, advantages, and limitations of these major methods are systematically compiled in Table 2. The following subsections will discuss each of these techniques in greater detail.

#### 3.6.1. Mass Spectrometry Analysis Technology

Mass spectrometry serves as a predominant analytical technique for structural characterization of proanthocyanidins. ESI-MS maintains molecular structural integrity via soft ionization. Coupled with tandem MS-derived fragmentation patterns, this approach enables precise determination of proanthocyanidin monomeric constituents and interflavan linkage configurations. For instance, ESI-MS analysis revealed that GC and EGC are the predominant subunits of SBPs [18]. To enhance the analytical throughput and detection sensitivity, ultra-high performance liquid chromatography-tandem mass spectrometry (UHPLC-MS/MS) can be used to accurately quantify proanthocyanidins in complex matrices [62]. Matrix-assisted laser desorption/ionization time-of-flight mass spectrometry (MALDI-TOF MS) is suitable for characterizing the polydispersity of high DP proanthocyanidins due to its wide molecular weight detection range.

#### 3.6.2. Nuclear Magnetic Resonance Technology

Nuclear magnetic resonance (NMR) spectroscopy is a critical method for the stereochemical elucidation of oligomeric proanthocyanidins (DP ≤ 4). One-dimensional proton nuclear magnetic resonance (^1^H/^3^C NMR) spectroscopy can determine the type of monomer and the C4 → C8 or C4 → C6 connection mode through differences in chemical shifts [63,64]. Two-dimensional heteronuclear single quantum coherence spectroscopy can precisely locate the proton-carbon correlation network and reveal the spatial configuration of the oligomer. However, as the DP increases, nuclear magnetic resonance spectroscopy faces three major technical bottlenecks: (i) instruments are still unable to completely resolve overlapping signals [65]; (ii) the structural complexity of proanthocyanidins increases significantly with increasing polymerization, making it more difficult to resolve them; (iii) the deuterium solvent needs to be improved to increase the solubility of the polymer [66]. Consequently, extant NMR studies have focused on oligomers with DP ≤ 10, and proanthocyanidins with DP > 10 are mainly inferred indirectly by mass spectrometry.

#### 3.6.3. Thiolysis Coupled Analysis Technology

Thiolysis-HPLC/MS enables structural elucidation of highly polymerized proanthocyanidin species intractable to conventional NMR characterization. The thiolysis reaction selectively cleaves specific chemical bonds in proanthocyanidins, generating thiol adducts that exhibit enhanced chromatographic separation performance and detection sensitivity. When coupled with high-performance liquid chromatography, this approach enables the separation and quantification of monomeric derivatives by HPLC, thereby analyzing the structural characterization of proanthocyanidins [67]. Specifically, the thiolysis method uses benzyl mercaptan to decompose proanthocyanidins, converting extension units into GC-benzyl thioether, EC-benzyl thioether, and other derivatives, while terminal units remain as free monomers. Therefore, the ratio of GC-benzyl thioether to EC-benzyl thioether can reflect the ratio of GC to EC in the extension units, but it does not include the terminal units. For polymers with ADP greater than 10, the proportion of terminal units is less than 10% of the total units, so the ratio of GC to EC in extension units may approximate the total GC/EC ratio of the entire molecule. In comparison with the use of ^13^C-NMR spectroscopy, the advantage of this method is that it can analyze components with ADP > 10, which appear as broad, unresolved peaks in the NMR spectrum [26].

## 4. Antioxidant Capacity

SBPs exhibit remarkable antioxidant capacity, arising from their distinctive structural attributes including their rare monomeric composition, predominantly comprising GC and EGC. This structural configuration confers SBPs with an enhanced density of antioxidant-active phenolic hydroxyl groups. Zhu et al. [30] demonstrated that SBPs exhibit a 4.8-fold enhancement in radical scavenging capacity compared to ascorbic acid at equimolar concentrations. Notably, combination index values <0.3 were observed across SBP/vitamin C molar ratios of 5:5–2:8, denoting pronounced synergistic antioxidant interactions. In addition, SBPs were found to have a considerable protective effect against oxidative damage to cells and tissues [15]. These results underscore the potential of SBPs as natural antioxidants for incorporation into food additives and underscore the necessity for further exploration of sea buckthorn as a health-promoting resource.

Specifically, the antioxidant capacity of SBPs can be reflected in their protective effect on mitochondrial membranes under conditions of oxidative stress and its neutralizing effect on lipid free radicals. In RAW264.7 macrophages exposed to H_2_O_2_, SBPs (100 μg/mL) restored mitochondrial membrane potential to 85% of the control level and inhibited the opening of the mitochondrial permeability transition pore. Furthermore, transmission electron microscopy revealed that the mitochondrial cristae structure remained intact in SBP-treated cells, in contrast to the vacuolated and swollen mitochondria observed in damaged cells not treated with SBPs. In addition, SBPs significantly reduced lipid peroxidation, lowering malondialdehyde levels by approximately 62% at 50 μg/mL [68].

## 5. Biological Activity

**Figure 3:**
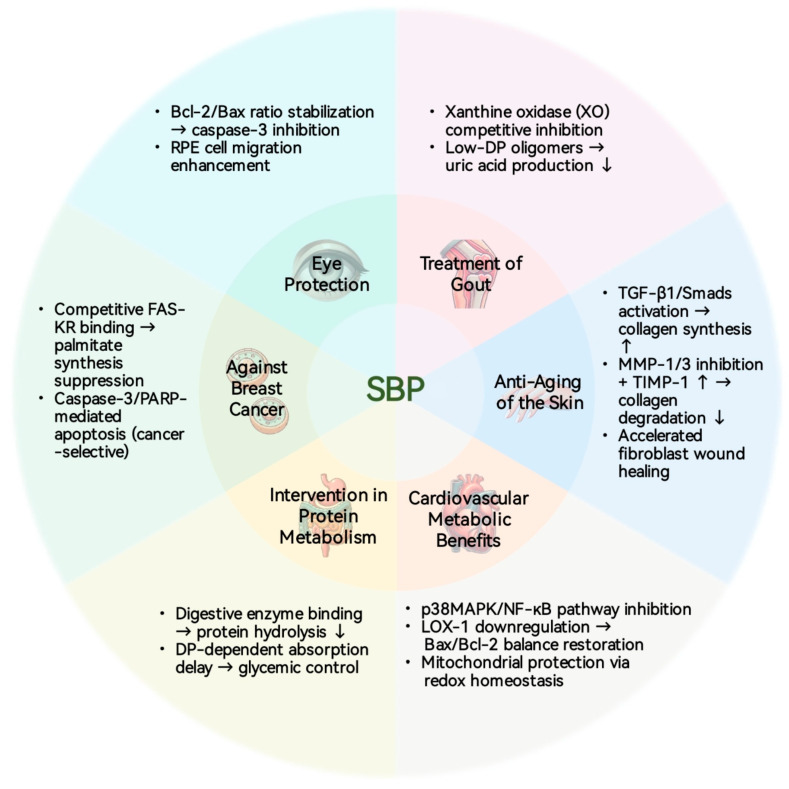
Biological activities of SBPs.

SBPs exhibit a spectrum of biological activities that highlight their potential as nutraceutical agents. Figure 3 provides a visual summary of these properties. To complement this visual summary with specific experimental context, Table 3 compiles key findings from the supporting literature. It presents the experimental models, mechanisms of action, and reported effective concentrations for each activity, allowing for a direct comparison of the current evidence. The following subsections will now explore these bioactivities in greater detail.

### 5.1. Cardiovascular and Metabolic Benefits

By targeting endothelial dysfunction, SBPs offer a promising approach to mitigate the risk of atherosclerosis (AS), a pathological process underlying major cardiovascular events such as coronary artery disease and ischemic stroke [59]. A study utilizing human umbilical vein endothelial cells (HUVECs) damaged by palmitic acid demonstrated that SBP treatment (100 μg/mL) restored mitochondrial membrane potential to 78.3% of baseline levels and reduced lactate dehydrogenase leakage by 42.7%, confirming a potent cytoprotective effect. This protection appears to be achieved through a dual mechanism, whereby SBPs mitigate oxidative stress through inhibition of the p38MAPK/NF-κB inflammatory pathway while also preventing apoptosis via downregulation of LOX-1 expression [20]. Although these in vitro findings provide a strong mechanistic rationale for SBPs’ protective role, their clinical relevance awaits confirmation from future in vivo studies in appropriate animal models.

### 5.2. Eye Protection

SBPs show potential in mitigating the progression of age-related macular degeneration (AMD), the leading cause of irreversible vision loss globally [60], by protecting retinal pigment epithelial (RPE) cells from oxidative stress. In an in vitro model of H_2_O_2_-induced damage, SBPs (50 μg/mL) restored RPE cell migration capacity to 85% of control levels, a critical process for retinal repair. This cytoprotective effect was attributed to its anti-apoptotic activity, demonstrated by the stabilization of the Bcl-2/Bax ratio and a 63% inhibition of caspase-3 activation [61]. While these cellular-level results are promising, their clinical translation requires further investigation into ocular bioavailability, optimal dosage, and advanced delivery strategies such as nanoparticle formulations [22,62]. Consequently, validation through in vivo models is a critical next step to establish the therapeutic efficacy of SBPs for promoting eye health.

### 5.3. Anti-Breast Cancer Activity

SBPs exhibit therapeutic potential against breast cancer, which remains the most frequently diagnosed malignancy in women globally [70]. SBPs exerts a substantial inhibitory effect on human breast cancer cells by selectively targeting fatty acid synthase (FAS), a pivotal enzyme overexpressed in many human cancers. Mechanistic studies have revealed that SBPs competitively bind to the ketoacyl reductase domain of FAS, with an IC_50_ value 29 times lower than that of the traditional inhibitor cerulenin, highlighting its superior potency. This inhibition of FAS effectively reduces palmitate synthesis, subsequently inducing apoptosis in MDA-MB-231 breast cancer cells through PARP cleavage and caspase-3 activation. Critically, SBPs did not induce apoptosis in normal breast cells, demonstrating a notable degree of cancer cell selectivity [21]. While this selectivity is a significant advantage, these preliminary in vitro findings require rigorous validation in animal models to assess both therapeutic efficacy and systemic safety before any clinical potential can be claimed.

### 5.4. Anti-Aging Skin Care

Skin aging remains a central focus in both dermatological research and consumer dermatology, driven by complex interactions between intrinsic and extrinsic aging pathways. Intrinsic aging, characterized by progressive biological decline over time, synergizes with extrinsic factors—notably chronic UV radiation exposure—to accelerate cutaneous aging processes [71]. Oxidative stress is a critical molecular mediator in this pathophysiology, where excessive reactive oxygen species generation disrupts dermal homeostasis and potentiates cutaneous senescence. This oxidative damage manifests through some hallmark clinical presentations like (i) loss of elastic fiber resilience, (ii) degradation of collagen-rich extracellular matrix architecture, and (iii) development of persistent rhytides [72].

Liu et al. [15] conducted a comprehensive study to evaluate the potential of SBPs, extracted from sea buckthorn, in mitigating skin aging through systematic in vitro and in vivo experiments. In their in vitro studies, human skin fibroblasts (HSFs) were exposed to H_2_O_2_ to induce oxidative stress, simulating skin aging conditions. Subsequently, the cells were treated with SBPs and analyzed for various endpoints. In the animal studies, a skin aging model was established by subcutaneous injection of D-galactose into the dorsal neck region of mice. SBPs were then administered to the mice via oral gavage, and its effects on skin tissue were evaluated. The combined results demonstrated that SBPs exhibit significant anti-skin aging activity both in vitro and in vivo through dual mechanisms involving antioxidant protection and collagen regulation [72]. Specifically, SBPs markedly enhance the activities of SOD and glutathione in skin cells, effectively scavenging excess reactive oxygen species (ROS) and thereby mitigating oxidative stress-induced damage to the skin. Additionally, SBPs promote the synthesis of type I collagen (Col I) by activating the TGF-β1/Smads signaling pathway while inhibiting Col I degradation through modulation of the matrix metalloproteinase and tissue inhibitor of metalloproteinase system, thus maintaining the stability of the extracellular matrix. Crucially, the anti-aging activity of SBPs stands out as the most robustly supported claim to date, as the mechanistic insights from cell cultures were directly corroborated by efficacy in an in vivo animal model. This progression from cellular to systemic evidence significantly elevates its translational potential compared to the other bioactivities discussed.

### 5.5. Treatment of Gout

SBPs offer a potential strategy for managing gout, a prevalent inflammatory arthritis affecting millions worldwide [73] and leading to chronic disability if left untreated [74], by directly inhibiting the pivotal enzyme xanthine oxidase (XO). Biochemical assays have demonstrated that SBPs competitively bind to the active site of XO, and this inhibitory activity is dependent on the DP. Specifically, oligomeric fractions (ADP = 3.7–5.7) exhibited 2.3 times the inhibitory efficiency of higher DP fractions (ADP = 8.5) in reducing uric acid production [28]. While the direct inhibition of a key enzyme in an in vitro assay provides a clear molecular target, confirming the clinical relevance of this finding requires in vivo studies to demonstrate a corresponding reduction in systemic uric acid levels.

### 5.6. Protein Metabolism Intervention

By inhibiting digestive enzymes, SBPs demonstrate potential as modulators of protein metabolism. This effect also demonstrates a dependence on the DP, as shown in in vitro experiments where high-DP SBPs (ADP = 10.6) reduced the hydrolysis rate of a BSA-pepsin complex by 58%, compared to a 32% inhibition by lower-DP SBPs (ADP = 5.6) [27]. This enzymatic inhibition suggests SBPs could function as a dietary ingredient to slow the absorption of protein degradation products. Such modulation of nutrient absorption offers a potential strategy for managing postprandial metabolic responses, which is critical for individuals with diabetes who face adverse health consequences from blood glucose fluctuations [75]. However, these in vitro findings establish a hypothesis that requires validation through human or animal feeding studies to confirm its physiological relevance.

## 6. Industrial Applications

Traditional sea buckthorn processing primarily involves juicing, which yields byproducts such as sea buckthorn seeds that are rich in phenolic compounds (approx. 7.3 g/100 g) and proanthocyanidins (approx. 3.4 g/100 g) [28]. However, these byproducts are typically discarded, resulting in considerable waste. Extracting SBPs from these seed residues offers a promising approach to optimize production chains and achieve high-value utilization of sea buckthorn byproducts, thereby promoting sustainable industrial practices. In particular, SBPs demonstrate industrial potential in food preservation and antimicrobial applications, cosmetic formulations, and nutraceutical development. These applications are contingent upon validated safety and efficacy profiles. For instance, evaluation of SBP cytotoxicity by Ma et al. [76] using ARPE-19 cells revealed no toxicity within the 0–1200 µg/mL concentration range. Specifically, concentrations of SBPs below 400 µg/mL did not significantly affect cell viability, while concentrations above 800 µg/mL promoted the proliferation of ARPE-19 cells, demonstrating a favorable safety profile for potential biomedical applications.

**Figure 4:**
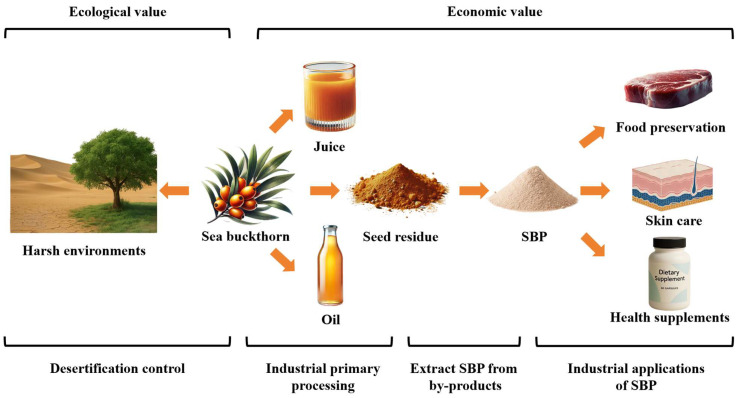
Economic and ecological value of sea buckthorn.

Figure 4 illustrates the ecological and economic value of sea buckthorn, with a specific focus on the industrial applications of SBPs.

### 6.1. Food Preservation

Proanthocyanidins are a class of naturally occurring antioxidants and antimicrobial agents capable of supplying phytonutrients to support a healthy diet while simultaneously extending the shelf life of food products. A research investigation assessed the efficacy of grape seed proanthocyanidins in preserving pork patties, demonstrating that the incorporation of 0.3% proanthocyanidins significantly diminished volatile basic nitrogen and 2-thiobarbituric acid reactive substances levels. This treatment also preserved the meat’s color and pH stability, thereby prolonging its shelf life under storage conditions at 4 °C. Moreover, in comparison to control samples, proanthocyanidins markedly suppressed the total bacterial count and *Escherichia coli* population. These results indicate that proanthocyanidins effectively mitigate oxidative degradation and microbial spoilage in meat, rendering them a viable option for industrial meat product manufacturing [77]. Blueberry-derived proanthocyanidins have similarly exhibited antimicrobial properties. A separate study explored their inhibitory effects against the foodborne pathogen *Cronobacter sakazakii*, revealing that a concentration of 5 mg/mL of blueberry proanthocyanidins reduced C. sakazakii strains 29004 and 29544 from initial counts of 8.25 ± 0.12 log CFU/mL and 8.48 ± 0.03 log CFU/mL, respectively, to undetectable levels within one hour [78]. Although specific studies remain limited, SBPs are hypothesized to serve as a promising source of antimicrobial agents. Michel et al. [79] conducted antimicrobial, antioxidant, and phytochemical analyses of sea buckthorn extracts, identifying that the water-extracted fraction, enriched with phenolic compounds, exhibited superior activity, particularly against *Bacillus cereus* and *Staphylococcus aureus*. The authors attributed this bioactivity to phenolic constituents in the seeds, notably proanthocyanidins.

Collectively, proanthocyanidins demonstrate notable antimicrobial and preservative capacities, positioning them as a natural reservoir of both antimicrobial and antioxidant agents. These properties suggest their potential as alternatives to synthetic preservatives in various applications, including food, cosmetics, and pharmaceuticals. Nevertheless, the specific preservative and antimicrobial attributes of SBPs warrant further exploration to substantiate their efficacy.

### 6.2. Skin Care

Research indicates that SBPs can mitigate oxidative stress by neutralizing free radicals, thereby protecting the skin from environmental damage. In addition to the previously noted capacity of SBPs to exhibit significant anti-skin-aging activity through the dual mechanisms of antioxidant protection and collagen regulation (as mentioned in Section 5.4), a separate study conducted in mice demonstrated that dietary grape seed proanthocyanidins can prevent UVB-induced skin tumors. The underlying mechanisms include enhanced DNA repair and stimulation of the immune system [80]. Furthermore, Zi et al. [81] investigated the effects of grape seed oligomeric proanthocyanidins on cultured human melanocytes following UV irradiation. Their findings revealed that these compounds significantly inhibit UV-induced melanogenesis in a dose-dependent manner by enhancing cell viability, scavenging intracellular ROS, regulating the cell cycle, and suppressing the protein expression of melanogenic enzymes, showing that proanthocyanidins may have a photoprotective effect in human melanocytes. These studies suggest that SBPs has potential applications in skincare.

### 6.3. Health Supplements

The structural variability of SBPs, arising from differences in raw materials, presents challenges for its application in precise disease treatment. However, its broad dosage safety range and rich bioactivity render it a promising candidate for use as a novel food additive and multifunctional health supplement. Internationally, grape seed proanthocyanidins have long been incorporated into dietary supplements. In comparison, SBPs possess a unique monomer composition and are similarly cost-effective, being derived as byproducts of sea buckthorn processing. These attributes strongly suggest SBPs’ potential as a popular novel dietary supplement.

## 7. Conclusions and Future Perspectives

Sea buckthorn is a multipurpose plant recognized for its ecological value in harsh environments and its traditional medicinal functions. The bioactivity of its fruit is largely attributed to SBPs, which have garnered significant scientific interest. A defining characteristic of SBPs is their unique chemical structure, distinguishing them from proanthocyanidins derived from other common sources like grape seed or berries. Specifically, SBPs are oligomers and polymers predominantly composed of highly hydroxylated (epi)gallocatechin units linked through B-type bonds. This distinct molecular architecture, hypothesized to be an evolutionary adaptation to stressors such as high altitude and intense UV radiation, provides the chemical foundation for their potent biological activities. The high degree of hydroxylation and planar configuration significantly enhance SBPs’ free radical scavenging, metal chelation, and anti-inflammatory capabilities. However, research into the extraction technology for these compounds is still in its nascent stages and necessitates further systematic investigation. While current approaches primarily rely on organic solvent-based methods, existing methodologies for proanthocyanidin extraction from other plant sources may offer valuable insights for process optimization. Structural analysis has shown that the biological activity of SBPs is closely related to its DP and monomer composition: oligomers exert antioxidant and anti-tumor effects by directly acting on target organs due to their high bioavailability, while polymers may indirectly affect metabolic health by regulating the intestinal flora. SBPs have demonstrated potential in the protection of cardiovascular health, the anti-aging of the skin, intervention in gout, and inhibition of breast cancer, and are gradually being applied in the development of functional foods, cosmetics, and health products, reflecting the modern transformation of the concept of medicine and food are of the same origin.

In light of these observations, future research should concentrate on the following areas: (i) Technological innovation and standardization. Further systematic investigations into SBP extraction methodologies are warranted, including parameter optimization of existing protocols and exploration of novel extraction techniques. (ii) In-depth mechanistic research. Multi-omics techniques will be integrated to reveal the cross-scale action network of SBPs, such as its mitochondrial protection, epigenetic regulation, and the mechanism of gut microbiota-host interaction. (iii) Exploring biological activities and pathways to industrialization. Deeper investigation into the broad spectrum of SBPs’ biological activities is essential to fully understand their potential. However, translating this potential into viable industrial applications requires overcoming significant practical barriers that currently represent critical knowledge gaps. Future research must therefore address not only the stability of SBPs in real food matrices, but also initiate foundational safety and toxicological assessments needed to navigate international regulatory frameworks. Addressing these technical and regulatory prerequisites is fundamental for the eventual commercialization of SBPs. (iv) A comprehensive industry chain for sea buckthorn will be established, and functional additives fortified with SBPs will be formulated using pomace to minimize production costs. The integration of ecological and economic benefits will be achieved through the promotion of sea buckthorn cultivation in conjunction with desertification control initiatives. The resilience of the regional economy will be bolstered by a linkage model of ecological restoration-resource development-health industry, thereby facilitating a mutually beneficial scenario for ecological protection and resource utilization.

## Figures and Tables

**Table 1:** SBP extraction/purification conditions and results.

Buckthorn Part	Extraction Method	Purification Method	Content	Purity	Characterization	Reference
Bark	Aqueous ethanol extraction (Temp: 21 °C, pH: 5.13, ethanol: 65%, Ratio: 1:10 *w*/*v*, Time: 90 min)	D3520 macroporous adsorption resin purification (Elution with 30% ethanol)	\	>95%		[25]
Seed	Water-acetone (3:7 *v*/*v*) extraction (3×, 2 h each), hexane wash	Sephadex LH-20 column chromatography (stepwise elution: H_2_O → ethanol → H_2_O/Acetone); further fractionation of polymer fraction	\	\	Characterized fractions (e.g., Fraction I: polymeric, ADP 12.2, 81.2% prodelphinidins)	[26]
Berry Pulp	Optimized: Acidified Acetone-Water (70:30 *v*/*v*, +1% acid), 3× extractions	Sephadex LH-20 column chromatography (Wash: 95% ethanol, Elute: 50% Acetone)	1.2% DW	66.2%	ADP 7.4	[27]
Seed Kernel	Same as above	Same as above	4.6% DW	70.4%	ADP 5.6	
Seed Coat	Optimized: Acidified Acetone-Water (60:40 *v*/*v*, +1% acid), 3× extractions	Same as above	0.9% DW	59.5%	ADP 8.2	
Leaves	Same as above	Same as above	0.6% DW	60.2%	ADP 10.6	
Seed	Defatted (hexane), then Methanol/Water (7:3 *v*/*v*) extraction (5×, 30 min each)	Ethyl acetate wash removes less polar phenolics, then Sephadex LH-20 column chromatography (Stepwise elution: water → 75% ethanol)	3.4% DW	68.6% (SPA-2)	ADP 14.7, Prodelphinidins 83.6%. Units: (Epi)Catechin and (Epi)Gallocatechin	[28]
Berry (Whole)	Acetone/Water/Acetic Acid (80:19.5:0.5 *v*/*v*) extraction (3×, 15 min sonication each)	Sephadex LH-20 column chromatography (Wash: Water; Elute Fractions: I: MeOH/Water 20:80, II: Acetone/Water 70:30, III: Acetone/Water 70:30). Fraction II used for analysis.	\	\	DP 2–11 detected (HILIC-MS). Only B-type. Main units: (Epi) Gallocatechin. Dimers 40%, Trimers 40%, Tetramers 20% (molar ratio of DP 2–4). The majority are higher polymers (HILIC-UV).	[24]
Berry Puree (seedless)	Same as above	Same as above	23.0–70.2 mg/100 g FW	\	Only B-type PAs detected. Main units: (Epi)Catechin and (Epi)Gallocatechin. DP 5–11 not detected significantly. Oligomers (DP 2–4) are 0.3–14.4% of Total PAs.	[29]
Berry (Whole, various origins)	Same as above	Sephadex LH-20 column chromatography (Wash: Water; Elute Fractions: I: MeOH/Water 20:80; II: Acetone/Water 70:30; Clean: MeOH)	0.39–1.94% DW	\	Only B-type PAs. DP up to 11 detected (HILIC-MS). Oligomers (DP 2–4) are 0.5–5% of total PAs. Significant differences in total PA and oligomer profiles exist between subspecies and locations.	[16]
Berry Powder (Qinghai)	Hot water extraction (1:15 *m*/*v*, 55 °C, 4 h, 2×)	AB-8 macro-porous resin enrichment (Elution: 30% ethanol), followed by spray drying	\	91.5%	LC-MS/MS identified dimers: (−)-epicatechin gallate, procyanidin B, (+)-gallocatechin-(+)-catechin, (+)-gallocatechin dimer. Low degree of polymerization (mostly dimers). UV_max_ 280 nm. Fourier-Transform Infrared Spectroscopy confirms structure.	[30]

**Table 2:** Comparison of major structural identification methods for SBPs.

Identification Method	Principle and Application	Key Information Provided	Advantages	Limitations	References
Mass Spectrometry (MS)	Structure identification via molecular and fragment ions. ESI-MS is suitable for oligomers; MALDI-TOF-MS is for high-DP polymers.	Monomer composition, linkage type (A/B-type), degree of polymerization (DP) distribution.	High sensitivity; capable of analyzing complex mixtures and high-DP polymers.	Difficulty in distinguishing stereoisomers; challenging for quantification.	[18,62]
Nuclear Magnetic Resonance (NMR)	Precise 3D structure elucidation by analyzing ^1^H and ^13^C chemical shifts and coupling constants.	Monomer type, linkage position (C4 → C8/C4 → C6), stereochemistry (2,3-cis/trans).	Provides definitive stereochemical information.	Low sensitivity, severe signal overlap; only suitable for purified, low-DP (<10) samples.	[63,64,65,66]
Thiolysis-HPLC/MS	Cleaves interflavan bonds using a nucleophilic reagent (e.g., benzyl mercaptan) to form derivatives for analysis.	Composition and ratio of extension vs. terminal units; calculation of average degree of polymerization (ADP).	Enables compositional analysis of high-DP polymers; relatively accurate for quantification.	Destroys the original structure; cannot provide sequence or stereochemical information.	[26,67]

**Table 3:** Summary of reported biological activities and mechanisms of SBPs.

Biological Activity	Study Model	Key Findings and Mechanism	SBP Concentration/Dose	Reference(s)
Cardiovascular Benefits	Palmitic acid-damaged Human Umbilical Vein Endothelial Cells (HUVECs)	Restored mitochondrial membrane potential; inhibited the p38MAPK/NF-κB pathway; downregulated LOX-1 expression to reduce apoptosis.	100 μg/mL	[20]
Eye Protection	H_2_O_2_-induced AMD model in human RPE cells	Restored cell migration capacity; stabilized the Bcl-2/Bax ratio; inhibited caspase-3 activation to reduce apoptosis.	50 μg/mL	[22,60,61]
Anti-Breast Cancer	Human breast cancer cells (MDA-MB-231)	Competitively inhibited fatty acid synthase (FAS); induced apoptosis via PARP cleavage and caspase-3 activation with selectivity for cancer cells.	IC_50_ ≈ 50 μg/mL	[21]
Anti-Aging Skin Care	H_2_O_2_-induced HSFs; D-galactose-induced mouse model	Enhanced SOD and GSH activities; activated TGF-β1/Smads pathway for collagen synthesis; inhibited the MMP system.	In vitro: 50–200 μM; In vivo: 50–200 mg/kg	[15,69]
Gout Treatment	Xanthine Oxidase (XO) inhibition assay	Competitively bound to the active site of XO to reduce uric acid production; low-DP fraction was more effective.	Oligomer: Ki ≈ 2.1 μM	[28]
Protein Metabolism Intervention	In vitro pepsin digestion model of BSA	Bound to protein and reduced its hydrolysis rate in a DP-dependent manner; high-DP fraction was more potent.	5–20 mg/mL	[27]

## Data Availability

No new data were created or analyzed in this study. Data sharing is not applicable to this article.

## List of Abbreviations

ADP	Average Degree of Polymerization
AMD	Age-Related Macular Degeneration
AS	Atherosclerosis
BSA	Bovine Serum Albumin
C	Catechin
Col I	Type I Collagen
DES	Deep Eutectic Solvents
DP	Degree of Polymerization
DW	Dry Weight
EC	Epicatechin
EGC	Epigallocatechin
ESI-MS	Electrospray Ionization Mass Spectrometry
FAS	Fatty Acid Synthase
FW	Fresh Weight
GC	Gallocatechin
HILIC-MS	Hydrophilic Interaction Liquid Chromatography-Mass Spectrometry
HILIC-UV	Hydrophilic Interaction Liquid Chromatography-Ultraviolet
HPLC	High-Performance Liquid Chromatography
HSFs	Human Skin Fibroblasts
HUVECs	Human Umbilical Vein Endothelial Cells
LC-MS/MS	Liquid Chromatography-Tandem Mass Spectrometry
MAE	Microwave-Assisted Extraction
MALDI-TOF-MS	Matrix-Assisted Laser Desorption/Ionization Time-of-Flight Mass Spectrometry
MS	Mass Spectrometry
NMR	Nuclear Magnetic Resonance
NP	Normal-Phase
ROS	Reactive Oxygen Species
RP	Reversed-Phase
RPE	Retinal Pigment Epithelial
SBPs	Sea Buckthorn Proanthocyanidins
SOD	Superoxide Dismutase
UAE	Ultrasound-Assisted Extraction
UHPLC-MS/MS	Ultra-High Performance Liquid Chromatography-Tandem Mass Spectrometry
UV	Ultraviolet
XO	Xanthine Oxidase
